# Understanding Stress-Related Behavioral Phenotypes: Report from the 1st International Neuroscience Summer School and the 11th International “Stress and Behavior” Conference

**DOI:** 10.1155/2008/543075

**Published:** 2008-11-18

**Authors:** J. L. LaPorte, V. M. Klimenko, A. V. Kalueff

**Affiliations:** Center for Physiology and Biochemical Research (CPBR), 6-5 Cheshskaya Street, Kiev 01042, Ukraine

## Abstract

The 1st International Neuroscience Summer School and the 11th International Multidisciplinary Neuroscience and Biopsychiatry Conference on Stress and Behavior were held in St. Petersburg, Russia, during May 9–20, 2008. The summer school gathered 30 talented young scientists from 15 countries worldwide, and was dedicated to different topics of behavioral neuroscience. Many interactive courses were provided on neuropharmacology, animal phenotyping, and biopsychology. The conference's excellent scientific and social program attracted almost 500 delegates from 40 countries from many areas of stress research. The eclectic interaction between medical doctors, basic scientists, psychologists, and students made for a productive and collaborative environment, which contributed greatly to the success of the school and conference.

## 1. FIRST INTERNATIONAL NEUROSCIENCE SUMMER SCHOOL

**Figure 1:**
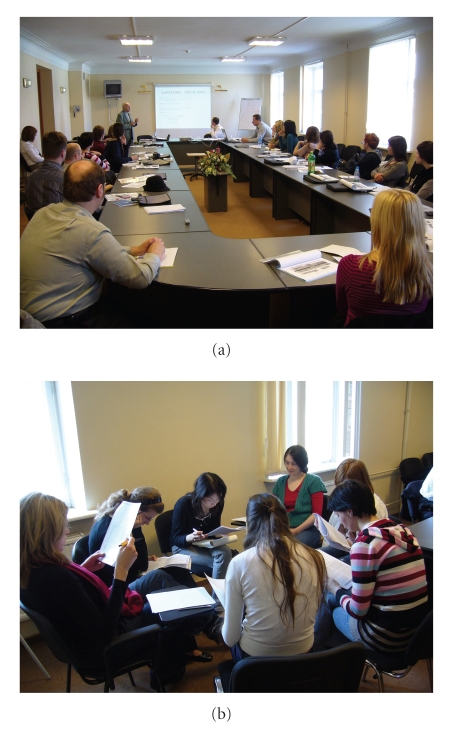
Scientific presentations and creative group discussions during the 1st International Neuroscience Summer School.

In May 9–15, the city of St. Petersburg, Russia, hosted the 1st International Summer School on the Behavioral Neuroscience and Neurogenetics of Stress. The school enjoyed a successful innovative debut, and attracted 30 students from 15 different countries. The students and faculty alike were stimulated through mutual interaction, brain-storming discussions, and were inspired by numerous lectures on topics such as biopsychiatry, neuropharmacology, animal phenotyping, and behavioral paradigms (Figure 1). These presentations were delivered by a team of lecturers that included Drs. Kalueff, LaPorte (USA), Lapin, Klimenko, and Tsikunov (Russia). Also, there were special workshops by Drs. Roelofs (Netherlands, Noldus Ethovision) and Liang (USA, CleverSystems Inc.) on new technological advances in the field in behavioral recognition. In addition to these scientifically oriented talks, the school’s program consisted of career development courses that covered topics such as scientific ethics, manuscript writing, manuscript submission, poster construction and presentation, and rebuttal letter writing. Several presentations from this part were kindly provided for the school courtesy of the American Physiological Society (APS).

The school participants also had the opportunity to visit the Institute of Experimental Medicine, where Ivan Pavlov spent a great portion of his productive career conducting research. During the visit, the group was provided with a tour of the institute, as well as demonstrations of the research currently being conducted in the institute’s laboratories. Among these were a model post traumatic stress disorder that utilized a python as the stress-inducing stimulus for rats, biofeedback technology for treating human social anxiety, and a “music of the brain” apparatus that produces music (for therapeutic biofeedback purposes) from the participants’ brainwave signals. As Pavlov spent some considerable time in St. Petersburg, the participants also toured his memorial flat and office.

## 2. THE 11TH “STRESS AND BEHAVIOR” NEUROSCIENCE CONFERENCE

**Figure 2:**
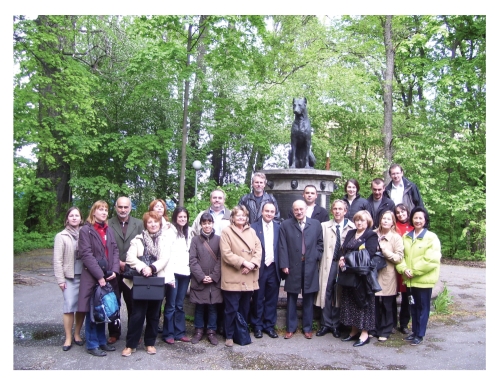
A group of conference speakers near the famous “Monument to the Dog” (commissioned by Ivan Pavlov) at the Institute of Experimental Medicine.

**Figure 3:**
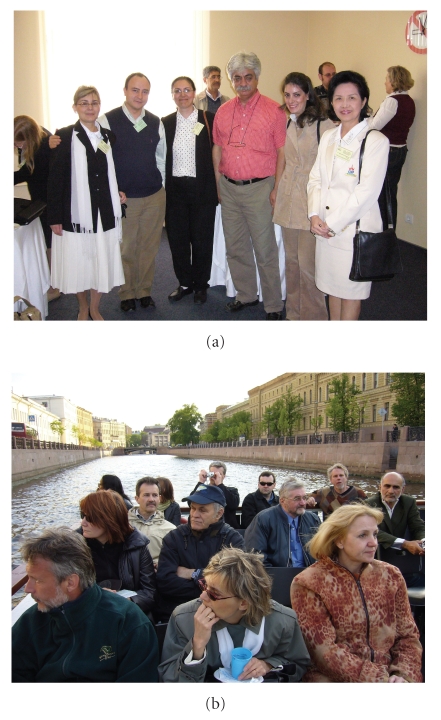
Conference delegates enjoying informal scientific discussions and the social program (during boat trip through the canals of St. Petersburg).

Immediately following the summer school, the 11th International Multidisciplinary Neuroscience and Biopsychiatry Conference on Stress and Behavior was held in St. Petersburg during May 16–20. The conference attracted almost 500 delegates (from 40 countries) representing different areas of stress research. The interaction between medical doctors, basic scientists, psychologists, and students made a productive environment for scientific discussions and collaborations (Figures 2 and 3).

The opening plenary lecture, dedicated to Hans Selye, was delivered by renowned neuropharmacologist Professor Izyaslav Lapin (Russia), who summarized his long-standing research on stress and neuro-kynurenines. Another plenary lecture on genetic factors in brain disorders was given by Professor John Quinn (UK). Genomics and polymorphisms composed the first symposium topic of the conference, chaired by Professor Quinn. Drs. G. Breen, A. MacKenzie (UK) and Professor H. Garner (USA) presented their recent data on genome-wide association studies of numerous psychiatric disorders.

The next symposium, chaired by Dr. Allan Kalueff (USA), focused on the tremendous progress that is being made in neuroscience research using animal models of brain disorders. During this symposium, Dr. A.R. Salomons (Netherlands) presented her research on indicators of anxiety phenotypes in certain inbred mouse strains based on aberrant habituation, and Dr. C. Tronche (France) discussed how memory retrieval in mice is altered in response to stress-induced rapid hippocampal corticosterone release. Professor V. Klenerova (Czech Republic) reported the behavioral effects of carbetocin on Wistar rats, and Dr. Zieba (Poland) described her recent behavioral studies exploring the role of corticotropin-releasing hormone (CRH) in rat frontal cortex.

The day 2 morning symposium on recent clinical issues in stress research was chaired by Professor Viktor Klimenko (Russia). Dr. V. Muhkin (Russia) presented innovative research on the variability of heart rates in patients of different mental states. There were also several interesting presentations on the effects of postwar stress and the correlating increase of mental and/or behavioral disorders in exposed children and adults. A special invited lecture after the symposium was given by Professor H. Garner (USA) on new software, developed in his laboratory to find repetition in the scientific literature and thereby discourage plagiarism in biomedical science. Another plenary lecturer, Professor N. Enginar (Turkey), focused on convulsive behaviors in fasted animals after antimuscarinic treatment and subsequent food intake. The third lecture was delivered by Dr. R. Czabak-Garbacz (Poland) on the behavior of women medical staff in stressful situations, giving the audience some excellent exposure to the realities of clinical practice.

The afternoon symposium of day 2 was focused on animal modeling of brain disorders. Professor Yuriy Pastuhov and Irina Ekimova (Russia) chaired this interesting set of presentations. Dr. Gyonos (Hungary) described the behavioral gender differences in animal models of depression, while Drs. T. Femenia, M.S. Garcia-Gutierrez, and J. Manzanares (Spain) gave 3 engrossing presentations on mouse neurogenetic and neurobehavioral phenotypes. One focused on the increases in anxiety-like behaviors in mice with deleted prodynorphin gene after exposure to restraint stress. Another talk reported that the overexpression of cannabinoid CB2 receptors lowered the emotional behaviors and impaired anxiolytic actions of benzodiazepines. The other talk presented research on the long-term effects of intracaudal administration of lactacystine on motor, emotional, and cognitive behaviors, as well as the expression of tyrosine hydroxylase in the basal ganglia.

The morning of day 3 welcomed Professor V. Klimenko (Russia) to speak on the topic of cytokines in the brain, followed by Dr. N. Wongwitdecha (Thailand) who spoke on how early social isolation in rats can interfere with the behavioral effects of ethanol. Progress in biological psychiatry research was the topic of the first symposium on that conference day. This symposium gave the audience a new look at some promising areas of research such as sleep deprivation and models of acute or chronic stress.

The afternoon symposium on day 3 was dedicated to stress research in clinical practice, and was cochaired by Dr. Juris Porozovs (Latvia), and Professor Viktor Klimenko (Russia). Several high-quality presentations were focused on areas of research such as psychiatric genetics, as well as novel therapeutic strategies for some brain disorders. One of the strategies of particular interest was presented by Dr. G.V. Manzhosova (Russia), who examined young children’s interactions with dolphins, and how this exposure can help improve the child’s stress coping. Conference lectures covered contemporary approaches to the diagnosis and treatment of children with attention deficit hyperactive disorder, given by Dr. Porozovs (Latvia). The impact of individual versus group housing on male mouse emotional behavior was emphasized by Dr. S.S. Arndt (Netherlands). The session finished with an inciting presentation by Dr. A. Kalueff on the domain interplay concept, and its utility for rethinking psychiatric genetics.

Recent advances in translational research were discussed in a day 4 morning symposium chaired by Dr. R. Czabak-Garbacz (Poland). Among the speakers was Dr. F. Netzer (France) who presented interesting research on the effect of direct stimulation of periaqueductal gray and cuneiforms nuclei in rat models, and their observations of inhibited cardiopulmonary cardiac reflexes. J. LaPorte presented a talk on a strategy for hybridizing animal modeling techniques. Professor S.H. Khakpour (Iran) presented her research on herbal extracts to treat depressive-like behavior in mice.

Research on cognitions and emotions were presented in a subsequent symposium chaired by Drs. Yuriy Pastuhov and Irina Ekimova (Russia). This symposium featured results from studies in the clinical field, as well as in other species such as mice and nonhuman primates. During the symposium, Dr. B. Adamcio (Germany) described the effect of prolonged social stress on prepulse inhibition in both individually and group house animals. Dr. O. Berchenko (Ukraine) spoke on her laboratories’ primate model, and gave a very interesting presentation on the influence of emotional stress on the cognitive processes of the primates. There was a presentation from Dr. Kozic (Serbia) on the application of brain and abdominal MRI in monitoring the effects of Wilson’s disease and understanding the consequences of its delayed recognition. Another presentation by Dr. A.C. Tsai (Taiwan) discussed the brain mechanisms of behavioral control in clinical subjects with varying anxiety levels.

Following this symposium, there was a series of lectures of high neuropharmacological interest. The first, given by Dr. I.V. Ekimova (Russia), was a look at the participation of gamma-aminobutyric acid (GABA) A receptors of the ventrolateral preoptic area in the somnogenic effects of heat shock protein HSP70. This was followed by a presentation from Dr. A.A. Lebedev on research where rats were gradually exposed to psychoactive drugs, examining CRH mRNA expression in hypothalamus and the amygdala. The last lecture was given by Professor T.N. Sollertinskaja (Russia) who spoke on the evolutionary peculiarities of brain functions, disturbed neuropeptide compensation and possible neurochemical mechanisms of stress across different species.

The final day 4 symposium, entitled “Towards integrative biological psychiatry,” was chaired by Professor Petr Shabanov (Russia) and featured Dr. B. Shultz-Klaus (Germany) speaking on the blocking of stress-induced behavioral changes in rats after a neurotoxic legion of the rostral peripheral cortex. Dr. E.V. Pushina (Russia) presented a talk on the relationships between neurons containing dopamine and nitric oxide synthase in the diencephalon of cyprinid teleosts. In addition, Dr. Barbier (France) reported the central effects of pyridostigmine treatment in chronically stressed rats, and Dr. A. Glystra (Greece) spoke on the use of benzodiazepines in emergency health centers in this country.

The day 5 of the meeting was traditionally held at the Institute of Experimental Medicine, a co-organizer of the conference. Morning plenary lectures were given by Professor Roger Pitman (Harvard University, USA) on interesting posttraumatic stress disorder (PTSD) twin studies of genetically identical siblings discordant for combat exposure, and by Dr. N.B. Lasko (USA), who presented recent data on the origins of psychiatric symptoms in PTSD. Professor V.M. Klimenko (Russia) overviewed the scientific history of Pavlov’s Department of Physiology, and Dr. O.E. Zubareva (Russia) focused on the role of interleukin 1b in learning processes. The conference’s final symposium was focused on the recent advances in psycho-immunophysiology. Among the speakers were Dr. M.N. Karpenko (Russia), who spoke on calpain expression in the central nervous system and peripheral cells associated with experimental allergic encephalomyelitis, and of Dr. I.N. Abdurasulova (Russia), who overviewed the interactions of stress and autoimmune disorders of the CNS.

In addition to multidisciplinary conference symposia, the scientific program also included two workshops highlighting new technologies in the field of behavioral registration. Dr. Roelofs (Netherlands), from Noldus Information Technology, presented on the Noldus Ethovision tracking system and its applicability to the goals of stress research. Dr. Liang (USA), representing CleverSystems Inc., demonstrated novel technologies in animal recognition. These workshops provided exposure to the cutting edge of neurobehavioral advances, and were of great benefit to the delegates.

The conference also had a rich array of social activities in addition to its scientific program (Figure 3). A boat trip through the canals of St. Petersburg, a guided bus tour of the major attractions of the city, a visit to Peterhof Royal Palace, and ballet in the magnificent Mariinsky Theatre were among the activities available for the conference’s delegates.

The conference was intended to foster interdisciplinary conversation and collaboration. Scientists came from all over the world to attend the meeting, resulting in a very diverse group of delegates. The audience was composed of roughly 60% psychiatrists and 40% neurobiologists, and included a large number of young scientists. As in the past, the organizing committee awarded a number of travel fellowships to support their participation. More information of these events can be found at the conference website at http://rus-neuroscience-soc.bm-science.com/stress-and-behaviour/.

Concluding the conference, it was announced that the forthcoming 12th International Neuroscience Conference “Stress and Behavior” will be held in St. Petersburg, Russia between May 16–20, 2009. The 2nd International Summer School on Behavioral Neuroscience of Stress will take place in Riga, Latvia (May 22–29, 2009), immediately after the conference.

